# Prophylactic wiring vs. non-wiring in hip arthroplasty: finite element and cadaveric analysis of proximal femur biomechanics

**DOI:** 10.1186/s42836-025-00331-0

**Published:** 2025-09-25

**Authors:** Atiwich Sangroungrai, Vorawit Atipiboonsin, Kamolsak Sukhonthamarn, Nattaphon Twinprai, Thewarid Berkban, Surasith Piyasin, Teerawat Laonapakul, Ong-art Phruetthiphat, Rit Apinyankul

**Affiliations:** 1 Department of Orthopaedics, Faculty of Medicine, Khon Kaen University, Khon Kaen, 40002 Thailand; 2 Department of Anatomy, Faculty of Medicine, Khon Kaen University, Khon Kaen, 40002 Thailand; 3 Department of Mechanical Engineering, Faculty of Engineering, Khon Kaen University, Khon Kaen, 40002 Thailand; 4 Department of Industrial Engineering, Faculty of Engineering, Khon Kaen University, Khon Kaen, 40002 Thailand; 5 Department of Orthopaedics, Phramongkutklao Hospital and College of Medicine, Bangkok, 10400 Thailand

**Keywords:** Prophylactic wiring, Periprosthetic femoral fracture, Finite element analysis, Proximal femur biomechanics, Cerclage wiring

## Abstract

**Background:**

Intraoperative periprosthetic femur fracture is a serious complication in hip arthroplasty, affecting patient outcomes. This study explored the biomechanical properties of the proximal femur, specifically comparing prophylactic cerclage wiring to non-wiring techniques using finite element analysis (FEA) and cadaveric biomechanical testing.

**Method:**

A finite element model of the proximal femur was constructed using Ansys software, allowing systematic assessment of both wiring area and technique to identify biomechanically optimal locations and configurations for cerclage placement. Twenty fresh cadaveric femurs were prepared according to standard protocols; the left femurs received cerclage wiring, while the right served as controls. Each femur was fitted with a femoral stem and tested under axial loading until catastrophic failure. Outcomes measured included ultimate load, seating load, subsidence distance, and energy absorption. Statistical analysis included the Shapiro–Wilk test for normality and independent t-tests for group comparisons.

**Results:**

The wiring group demonstrated comparable biomechanical performance to the non-wiring group across all measured parameters. Energy absorption was similar between groups (41.9 ± 18.1 Nm vs. 41.0 ± 19.1 Nm, *P* = 0.918). No significant differences were observed in ultimate load (7.6 ± 2.1 kN vs. 7.7 ± 2.0 kN, *P* = 0.901) or seating load (3.1 ± 0.7 kN vs. 3.4 ± 1.4 kN, *P* = 0.589). Similarly, subsidence distance showed no intergroup difference (7.7 ± 2.6 mm vs. 7.7 ± 3.8 mm, *P* = 0.978).

**Conclusion:**

Cerclage femoral wiring for prophylactic purposes during hip arthroplasty does not confer a significant biomechanical advantage over non-wiring techniques.

## Introduction

Intraoperative periprosthetic femur fracture is a recognized complication that can occur during total hip arthroplasty (THA). The incidence of this complication varies depending on the type of THA procedure. It has been reported to have a low incidence of 0.1%–1% for primary cemented THA and a high incidence of 5.4% for primary uncemented THA [1, 2].

To reduce the incidence of intraoperative periprosthetic femur fractures (PFFs), especially in patients at risk for intraoperative periprosthetic femur fracture [5–7], a preventive method involving the application of prophylactic cerclage (wire loop) around the proximal femur has been suggested and provides outstanding resistance to both axial and rotational stresses on the implant stem [3–5]. This approach aims to increase the resistance of the proximal femur to hoop stress, ultimately reducing the likelihood of crack formation and stem subsidence [4, 6, 7]. Prophylactic wiring has also been shown to be effective in preventing postoperative PFFs by increasing the resistance of the implant to rotation and improving its load to failure, particularly in the case of well-fix press-fit femoral implants [5].

The objective of this study was to examine stress resistance and sharing in the proximal femur during broaching and assess the effect of prophylactic cerclage wiring to prevent femoral neck calcar fractures in fresh cadaveric bones. We hypothesized that prophylactic wiring would result in a statistically significant difference in ultimate load compared with that of the control group. Additionally, we aimed to compare the subsidence distance of the stem and energy absorption of the proximal femur between bones with wiring and those without wiring when fractures occurred.

## Method

Ethical approval for the study and exemption from obtaining informed consent were approved by the Institutional Review Board (approval NO. HE671112). We first conducted a finite element analysis to identify the optimal location and configuration for prophylactic cerclage wiring of the proximal femur, aiming to prevent periprosthetic fractures, as informed by prior research. Subsequently, biomechanical tests were performed using cadaveric femoral bone to compare outcomes between femurs treated with cerclage wiring and those without.

### Finite element analysis (FEA) and determination of cerclage femoral wiring techniques

**Fig. 1:**
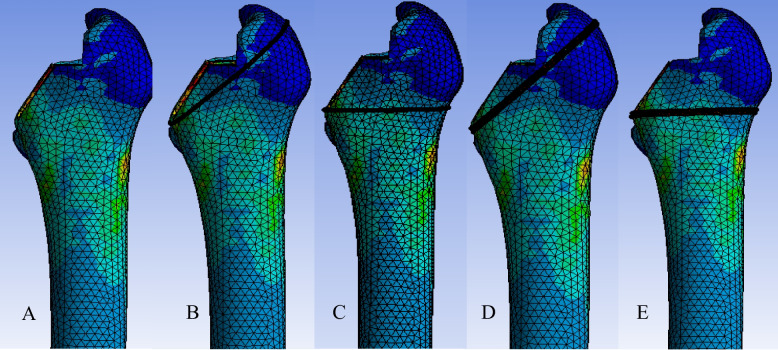
Finite element models of the different wiring techniques: **A**. Non-wiring; **B**. One single-loop wiring placed perpendicular to the femoral neck axis (one single-loop PN); **C**. One single-loop wiring placed perpendicular to the femoral shaft axis (one single-loop PF); **D**. One double-loop wiring placed perpendicular to the femoral neck axis (one double-loop PN); **E**. One double-loop wiring is placed perpendicular to the femoral shaft axis (one-double-loop PF). PN: wiring perpendicular to the femoral neck; PF: wiring perpendicular to the femoral shaft

**Fig. 2:**
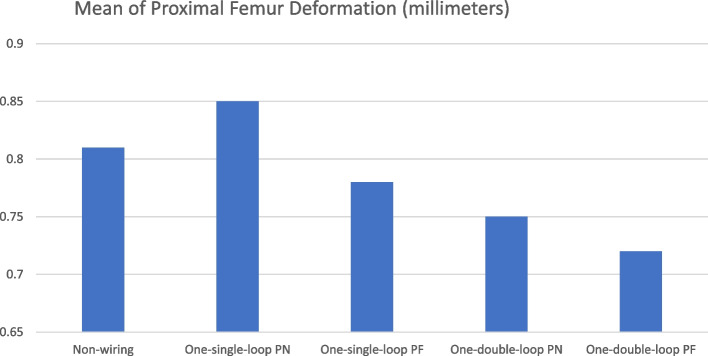
Mean deformation of the femoral calcar based on finite element analysis for different wiring techniques and wire placement locations. PN: wiring perpendicular to femoral neck; PF: wiring perpendicular to femoral shaft

To investigate potential fracture mechanisms, we conducted a computational simulation of the broaching process. A clinically representative femur with Dorr type B morphology was selected from our institutional anatomical imaging database, comprising a curated collection of femur CT scans. Using ANSYS finite element analysis software, we modeled the mechanical loading conditions during broaching under physiological parameters. Data generated from this analysis were comprehensively evaluated to compare deformation of the proximal femur across different techniques: non-wiring, single-loop wiring, and double-loop wiring. Additionally, two wiring positions were assessed: wiring perpendicular to the femoral neck axis (PN) and wiring perpendicular to the femoral shaft axis (PF), both situated just above the lesser trochanter (Fig. 1). In this phase, results from the finite element simulations (Fig. 2) were scrutinized to identify the wiring technique and position that provided the most biomechanically favorable wiring configuration, as indicated by least calcar deformation, for further biomechanical evaluation in fresh cadaveric femurs. Subsequent mechanical testing focused on measuring ultimate load to failure and energy absorption, comparing outcomes between femurs prepared with and without prophylactic wiring. This approach, integrating finite element modeling with targeted biomechanical validation, offers a robust framework for determining the most advantageous wiring technique and position for minimizing proximal femoral damage during broaching.

### Cadaveric femoral bone preparation

**Fig. 3:**
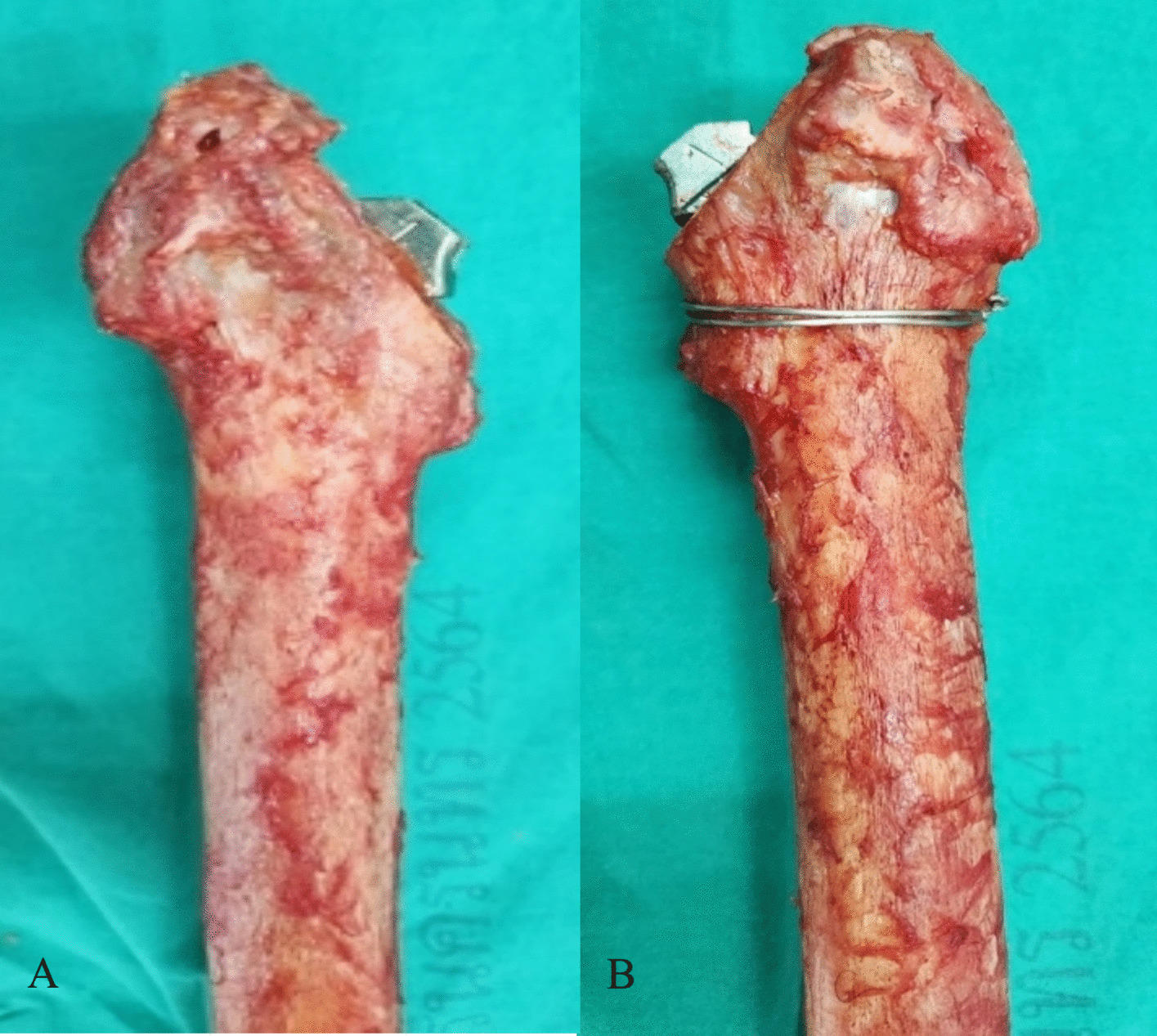
Fresh cadaveric femur after preparation with the manual technique: **A**, non-wiring; **B**, one double-loop wire (perpendicular to femoral shaft)

Fresh cadavers aged over 50 years were included in the study. Soft tissue was resected from the femoral bones, which were then screened with fluoroscopy. Pairs of bones classified as Dorr type B or C, based on established radiographic morphology criteria, were selected for inclusion (the study inclusion criteria were age over 50 years and Dorr classification B or C) [8]. Bones were excluded if there was evidence of prior trauma to the proximal femur, congenital deformity or disease, or any bone tumor of the femur as identified by fluoroscopy. This selection process was designed to ensure uniformity in sample characteristics and to properly assess bone quality. The femoral neck was osteotomized 10 mm above the lesser trochanter. Proximal femoral preparation was performed manually by a single fellowship-trained arthroplasty surgeon until an appropriately sized Zimmer Avenir stem, templated from fluoroscopic images, was achieved. The stem was then removed. On each left femur, cerclage wiring was applied using a 1.6 mm stainless steel (316L) wire in a one double-loop fashion, placed perpendicular to the femoral shaft axis (PF), just above the lesser trochanter, by manual technique. (Fig. 3).

### Mechanical testing

**Fig. 4:**
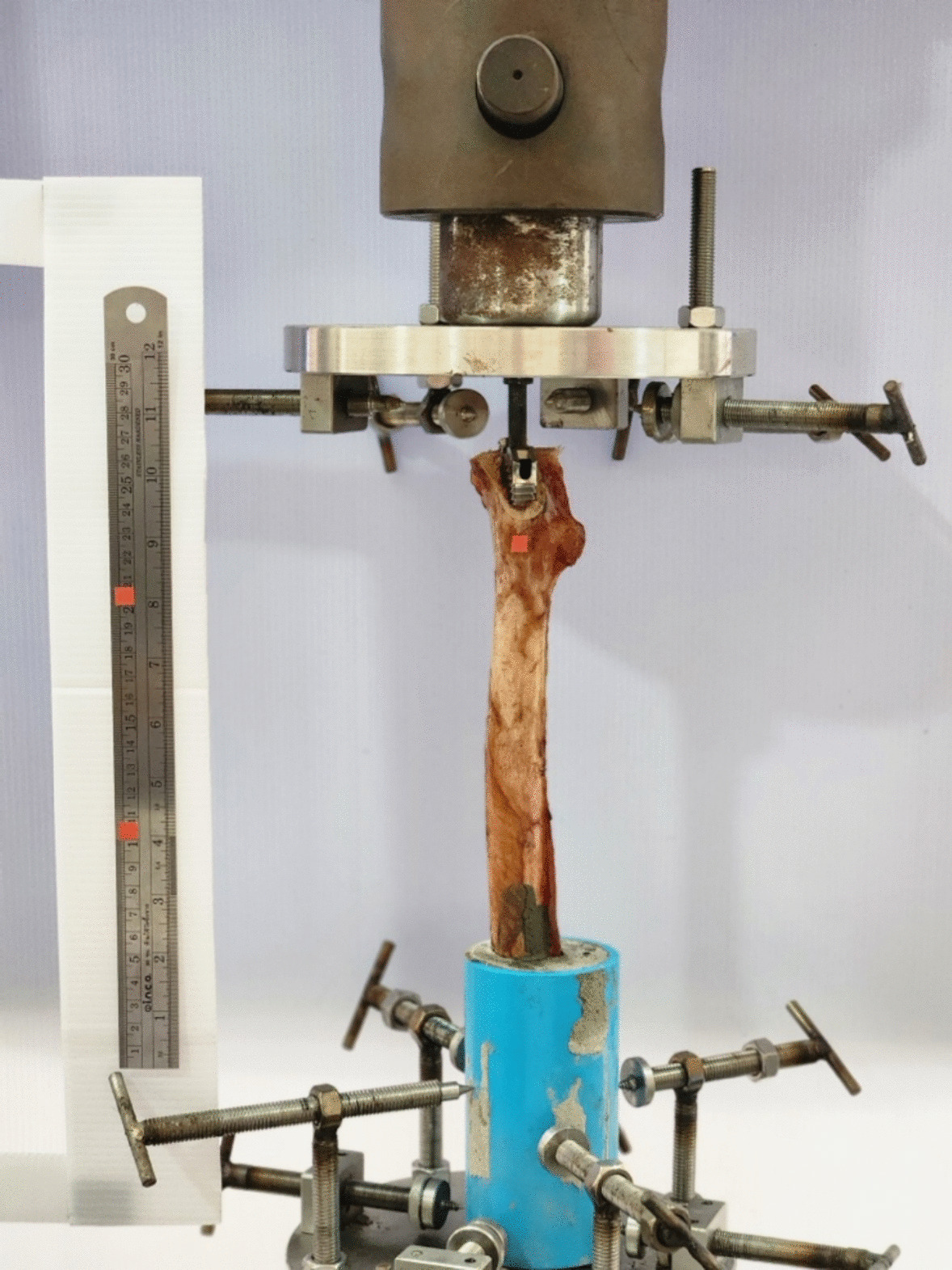
Biomechanical test setup: The specimen was positioned vertically on the table of a materials testing machine, and a hydraulic metal block of a servohydraulic testing system (Servopulser: SHIMADZU) applied a vertical load onto the prosthesis

Each cadaveric femur, implanted with a previously prepared, appropriately sized femoral stem, was subjected to mechanical testing using a hydraulic testing system with a maximum load capacity of 100 kN (Fig. 4). To standardize testing conditions, each femur was preloaded to 100 N for 30 s before being loaded in axial compression at a displacement rate of 1 mm/s until catastrophic failure. The service was provided by the Research Instrument Center, Khon Kaen University, Thailand cooperation with the Faculty of Engineering, Khon Kaen University.

**Fig. 5:**
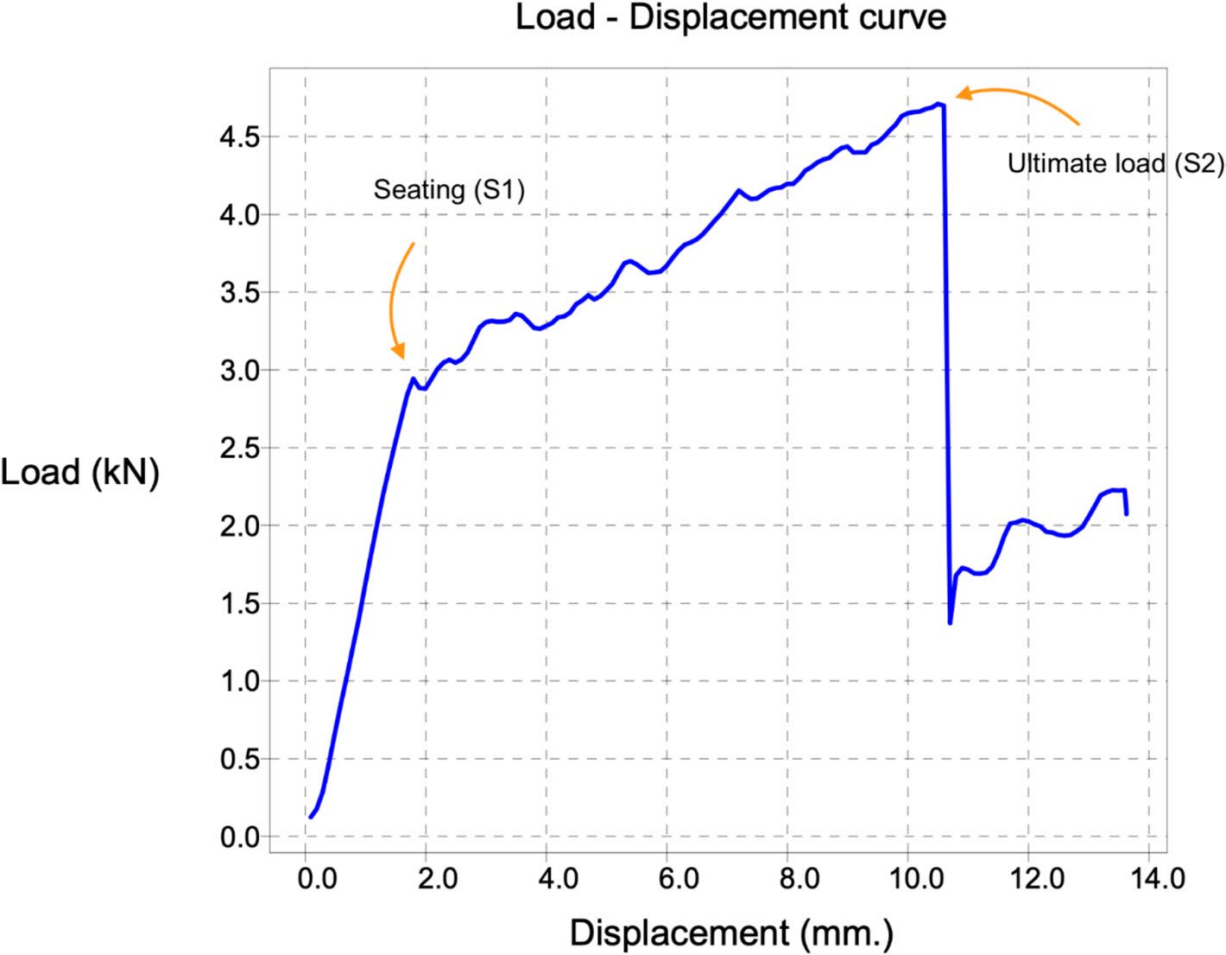
Load–displacement curve characteristics: The seating load refers to the load where the broaching instrument was first seated in the proximal femur and a change in slope was observed in the load–displacement graph, the ultimate load represents the maximum load at just before fracture occurs, subsidence denotes the displacement measured from the onset of seating load until failure, and energy absorption is quantified as the area under the curve from seating load to failure. kN: kilonewton; mm: millimeter

Each cadaveric femur was assigned to either the wiring or non-wiring group. Biomechanical parameters, including seating load, ultimate load, subsidence distance, and energy absorption, were analyzed from the load–displacement curve, as illustrated in Fig. 5. The area under the curve was calculated to determine energy absorption. This study aims to evaluate the effectiveness of prophylactic wiring in stabilizing the proximal femur. Additionally, statistical analyses were conducted to assess differences between the two groups and determine correlations between biomechanical parameters. These findings will enhance the understanding of prophylactic wiring’s impact on the biomechanical response of the proximal femur.

### Statistical analysis

Statistical analysis was conducted using SPSS software. All data are presented as means ± standard deviations (SDs). The mean ultimate loads between the wiring and non-wiring groups were compared using a two-sided Student’s t-test. A *P*-value of less than 0.05 was considered statistically significant.

## Results

The demographic characteristics of the fresh cadaveric femurs are presented in Table 1. This study included twelve femurs classified as Dorr type B and eight as Dorr type C, based on the Dorr classification, which assesses proximal femoral bone quality and morphology [8].

**Table 1:** Demographic characteristics of fresh cadavers and proximal femurs

Factors	N (%)/mean ± SD
Gender	
• Male (n)	1 (10%)
• Female (n)	9 (90%)
Age (mean ± SD) (years)	71.8 ± 8.6
Dorr type	
• B (n)	12 (60%)
• C (n)	8 (40%)

SD: standard deviation

Finite element analysis results for the various wiring techniques are illustrated in Figs. 1 and 2. Prophylactic femoral wiring using the one-double-loop technique in the optimal position—just above the lesser trochanter and perpendicular to the femoral shaft axis (one-double-loop PF)—demonstrated the greatest resistance to hoop stress, evidenced by the lowest mean proximal femur deformation in the analyses, 0.72 ± 0.24 mm, compared to other constructs of wiring (non-wiring 0.81 ± 0.23 mm, one-single-loop PN 0.85 ± 0.30 mm, one-single-loop PF 0.78 ± 0.25 mm, and one-double-loop PN 0.75 ± 0.23 mm).

**Fig. 6:**
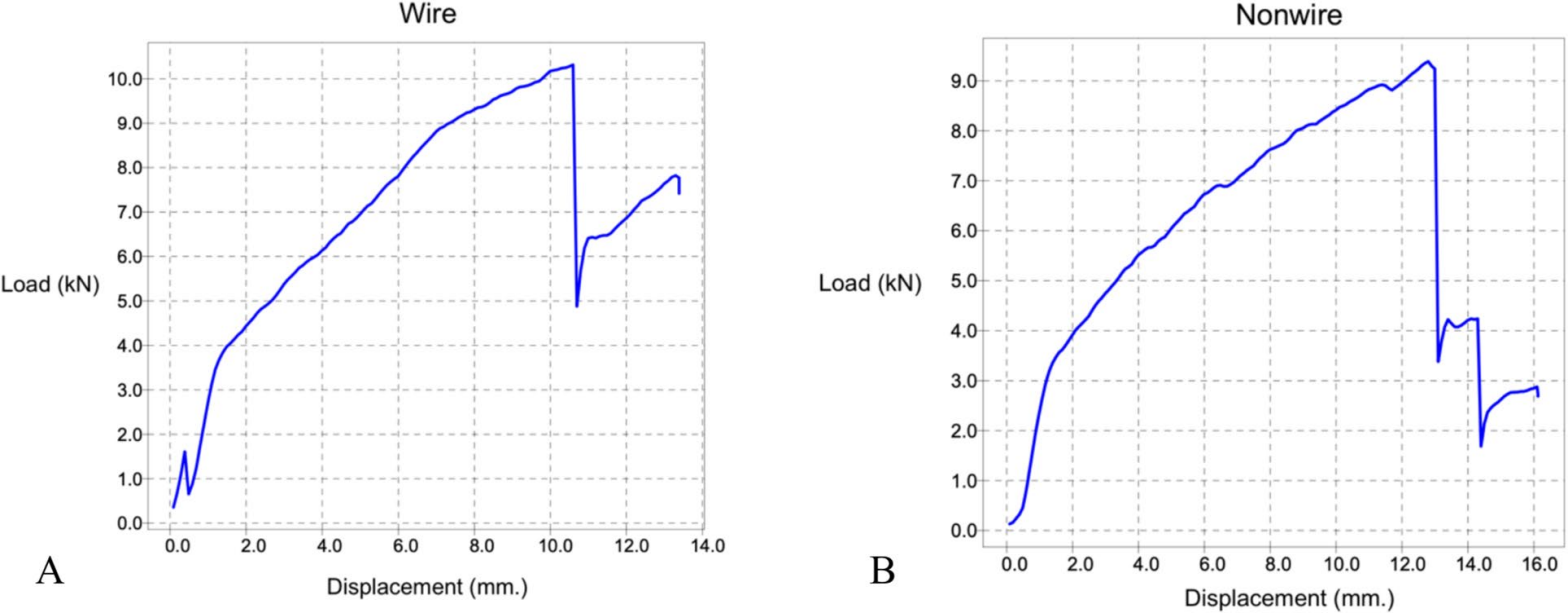
Load–displacement curves comparing femoral bones with (Curve A) and without (Curve B) cerclage wire fixation. Curve A represents the wired group and shows continued load resistance after reaching ultimate load, indicating residual stability post-fracture. Curve B represents the non-wired group and shows a sharp drop in load, suggesting immediate instability after fracture. Curve labels (A and B) are indicated directly on the graph for clarity. kN: kilonewton; mm: millimeter

Given the limited availability of cadaveric specimens for biomechanical testing, we selected the most promising configuration from the finite element analysis—the one-double-loop PF technique—for further experimental validation. At peak ultimate load, the wiring group demonstrated energy absorption of 41.9 ± 18.1 Nm, showing no statistically significant difference from the non-wiring group's absorption of 41.0 ± 19.1 Nm (*P* = 0.918). The ultimate load—defined as the maximum load at just before fracture occurs—and seating load—identified at the load where the broaching instrument was first seated in the proximal femur and a change in slope was observed in the load–displacement graph (Fig. 5)—were 7.6 ± 2.1 kN and 3.1 ± 0.7 kN, respectively, for the wiring group. These values did not significantly differ from the non-wiring group, which recorded 7.7 ± 2.0 kN (*P* = 0.901) and 3.4 ± 1.4 kN (*P* = 0.589). Subsidence distances were likewise similar: 7.7 ± 2.6 mm in the wiring group versus 7.7 ± 3.8 mm in the non-wiring group (*P* = 0.978) (Table 2). The cerclaged wire of femoral bone demonstrates a change in slope after reaching the ultimate load, indicating that the construct continues to resist displacement beyond initial fracture, consistent with improved post-fracture stability. Whereas, the non-cerclage wire of femoral bone displays an immediate drop in load following the ultimate load, reflecting rapid further displacement due to fracture propagation or crack extension, and indicates a lack of residual stability after failure. Load–displacement curves of femoral bones with and without cerclage wire fixation are shown in Fig. 6.

**Table 2:** Comparison of ultimate load, seating load, subsidence and energy absorption between wiring and non-wiring groups

**Variables**	**Mean** ± **SD**		***P-*value**
	**Wiring**	**Non-wiring**	
Ultimate load (kN)	7.6 ± 2.1	7.7 ± 2.0	0.901
Seating load (kN)	3.1 ± 0.7	3.4 ± 1.4	0.589
Subsidence (mm)	7.7 ± 2.6	7.7 ± 3.8	0.978
Energy absorption (Nm)	41.9 ± 18.1	41.0 ± 19.1	0.918

kN: kilonewton; mm: millimeter; Nm: Newton meter

Therefore, there were no significant differences between the wiring and non-wiring groups regarding ultimate load, seating load, subsidence distance, or energy absorption.

## Discussion

A satisfactory outcome is achieved by preventing intraoperative fractures of the proximal femur and minimizing associated complications. In practice, conversion to a cemented technique with risk assessment is most commonly employed when complications arise. However, the use of bone cement may increase the risk of other complications, such as fat or cement embolism, particularly in elderly patients with comorbidities [9–11]. Furthermore, managing cement-related complications can be challenging, which contributes to many surgeons’ preference for cementless techniques. This study aimed to evaluate the efficacy of prophylactic wiring of the proximal femur in preventing intraoperative fractures, particularly in bones at elevated risk [12, 13].

Differences among wiring techniques and positions were assessed using finite element analysis, which revealed that wiring just above the lesser trochanter, perpendicular to the femoral shaft axis (PF), resulted in the least deformation of the femoral calcar. The one-double-loop technique demonstrated greater strength than the one-single-loop technique by reducing proximal femoral calcar deformation. These findings align with earlier research indicating that the one-double-loop technique offers superior fixation compared to both the one-single-loop and two-single-loop techniques, particularly when placed perpendicular to the shaft axis [4, 14, 15]. This suggests that positioning the wiring perpendicular to the shaft and employing a double-loop configuration provides enhanced biomechanical strength.

A major strength of this study is the use of fresh cadaveric femora, which best approximate the biomechanical properties of living bone. Each specimen underwent pre-test templating comparable to clinical practice, ensuring appropriate fit of the prosthetic stem. However, biomechanical testing indicated no significant differences between the wiring and non-wiring groups with respect to energy absorption, ultimate load, seating load, or subsidence distance. Both groups absorbed no significant difference amounts of energy (wiring: 41.9 ± 18.1 Nm; non-wiring: 41.0 ± 19.1 Nm), and ultimate and seating loads showed no significant difference between groups (wiring: 7.6 ± 2.1 kN and 3.1 ± 0.7 kN; non-wiring: 7.7 ± 2.0 kN and 3.4 ± 1.4 kN). While no statistically significant difference in ultimate load was found between groups (*P* = 0.901), post hoc analysis revealed a negligible effect size (Cohen’s d = 0.035) with critically low statistical power (~ 5%). These findings suggest that the study was likely underpowered to detect small between-group differences, and thus, the null result should be interpreted cautiously. These biomechanical outcomes contrast with some previous studies, which found that prophylactic one-double-loop wiring significantly improved resistance to hoop stress (S. Wongsak et al.) [15]. The differing elastic properties between cadaveric and composite bone models may explain the variation in findings. In this study, wiring performed with the one-double-loop technique at the optimal position improved resistance to hoop stress, but this improvement was not statistically significant relative to the non-wiring group. Although no significant differences were observed in ultimate load, energy absorption, seating load, or subsidence distance, there were trends in the load–displacement patterns suggesting that wiring may influence construct behavior at higher loads. These results, alongside other reports, suggest that wired specimens may be less prone to comminuted or displaced fractures and could reduce the need for reoperation if fractures are undetected during surgery [5].

In addition to prophylactic wiring, femoral stem design—particularly stem length—has also been shown to influence the risk of intraoperative periprosthetic fractures. Registry and clinical studies have reported lower fracture rates with short or ultra-short cementless stems compared to standard-length designs, likely due to less invasive canal preparation and more favorable load transfer in osteoporotic bone. Staunton et al. found an acute fracture rate of only 0.6% among 3,192 short-stem THAs [16], while Luger et al. reported similarly low early fracture rates (< 1%) in short-stem implants using a minimally invasive anterolateral approach [17]. Furthermore, Melišík et al. demonstrated mid-term outcomes of the Proxima ultra-short anatomical stem in 130 patients, reporting only two perioperative fractures (1.5%) and excellent radiographic (98.5%) and clinical (100%) survivorship at nearly 10 years[18]. These findings reinforce the importance of stem selection as an adjunct to surgical technique in reducing fracture risk during cementless total hip arthroplasty.

While prophylactic wiring using the one-double-loop technique in the optimal position may enhance resistance to hoop stress, no statistically significant advantage was demonstrated in this study compared to non-wiring, potentially due to a combination of biomechanical and methodological factors. Notably, wiring may alter the proximal femur biomechanics, which may influence load distribution on the proximal cortex, femoral stem, and overall biomechanics [14].

### Limitations

Our study had several limitations: the absence of soft tissue in biomechanical tests, variability in cadaver bone properties, differences between the servo-hydraulic testing conditions and intraoperative impaction forces, and the predominance of female specimens in the sample. Several factors may contribute to the test results. First, the effects of static loading differ from those of dynamic impaction forces during surgery. Second, the shape of the femoral stem may not produce uniform hoop stress across the proximal cortex, potentially limiting the effectiveness of prophylactic wiring even when hoop stress resistance is increased. Third, the small sample size resulted in limited statistical power to detect subtle intergroup differences. This reduced sensitivity may explain the nonsignificant findings observed, warranting further investigation with a larger sample size. Fourth, our study relied on an FEA of a single Dorr type B femur. This single FEA specimen approach restricts the generalizability of our findings to other bone morphologies (e.g., Dorr types A and C) or to patients with varying bone quality, such as osteoporotic or unusually dense bone. Furthermore, because the FEA of this specimen guided the selection of wiring techniques for validation, the results of the subsequent biomechanical tests may be biased toward a single anatomical model.

## Conclusion

Cerclage femoral wiring for prophylactic purposes during hip arthroplasty does not confer a significant biomechanical advantage over non-wiring techniques.

## Data Availability

No datasets were generated or analysed during the current study.
